# Prevalence and Genetic Diversity of Avipoxvirus in House Sparrows in Spain

**DOI:** 10.1371/journal.pone.0168690

**Published:** 2016-12-22

**Authors:** Jorge Ruiz-Martínez, Martina Ferraguti, Jordi Figuerola, Josué Martínez-de la Puente, Richard Alexander John Williams, Amparo Herrera-Dueñas, José Ignacio Aguirre, Ramón Soriguer, Clara Escudero, Michaël André Jean Moens, Javier Pérez-Tris, Laura Benítez

**Affiliations:** 1 Departamento de Microbiología III, Facultad de Biología, Universidad Complutense de Madrid, Madrid, Spain; 2 Estación Biológica de Doñana, Consejo Superior de Investigaciones Científicas, Sevilla, Spain; 3 CIBER Epidemiología y Salud Pública (CIBERESP), Spain; 4 Departamento de Zoología y Antropología Física, Facultad de Biología, Universidad Complutense de Madrid, Madrid, Spain; Universidad de Granada, SPAIN

## Abstract

*Avipoxvirus* (APV) is a fairly common virus affecting birds that causes morbidity and mortality in wild and captive birds. We studied the prevalence of pox-like lesions and genetic diversity of APV in house sparrows (*Passer domesticus*) in natural, agricultural and urban areas in southern Spain in 2013 and 2014 and in central Spain for 8 months (2012–2013). Overall, 3.2% of 2,341 house sparrows visually examined in southern Spain had cutaneous lesions consistent with avian pox. A similar prevalence (3%) was found in 338 birds from central Spain. Prevalence was higher in hatch-year birds than in adults. We did not detect any clear spatial or temporal patterns of APV distribution. Molecular analyses of poxvirus-like lesions revealed that 63% of the samples were positive. Molecular and phylogenetic analyses of 29 DNA sequences from the fpv167 gene, detected two strains belonging to the canarypox clade (subclades B1 and B2) previously found in Spain. One of them appears predominant in Iberia and North Africa and shares 70% similarity to fowlpox and canarypox virus. This APV strain has been identified in a limited number of species in the Iberian Peninsula, Morocco and Hungary. The second one has a global distribution and has been found in numerous wild bird species around the world. To our knowledge, this represents the largest study of avian poxvirus disease in the broadly distributed house sparrow and strongly supports the findings that Avipox prevalence in this species in South and central Spain is moderate and the genetic diversity low.

## Introduction

Many wildlife species are reservoirs of pathogens and may transmit infectious agents to sympatric domesticated species (“reverse spill-back”) or endangered wildlife species or humans [1]. Managing the risk of further disease emergence requires improved understanding of the diversity and prevalence of circulating pathogens in natural populations, and the complexity of these multifaceted relationships.

Avian pox (APV) is a viral infection that causes proliferative lesions in wild and domestic bird species worldwide. Poxvirus infections are commonly cutaneous, though they may also produce virulent diphtheritic forms. The disease exists at very low prevalence where it is endemic and it has little impact on affected birds [2]. However the infection can cause economic losses in domestic poultry and the introduction of APV, and other pathogens, to remote island archipelagos (e.g. Galapagos and Hawaii) has caused dramatic declines to the immunologically naïve, not to mention rare, avifauna of those island groups[3–5]. The disease is caused by *Avipoxvirus*, a genus of enveloped double-stranded DNA viruses. Transmission is mainly via arthropod vectors, though it can also occur via respiratory aerosols or contact with infected birds or contaminated surfaces like perches or nests [2]. Poxvirus is extremely resistant to desiccation, and can survive in the environment for long periods [2] which may facilitate its transmission.

The International Committee on Taxonomy of viruses (ICTV) recognises 10 species in the genus *Avipoxvirus* [6], all named according to the first host where they were described. Phylogenetic analysis based on polymorphism of both P4b (major core protein) and DNA polymerase genes indicate the existence of three major clades, A (Fowlpox-like viruses), B (Canarypox-like viruses) and C (Psittacinepox-like viruses), and some recently described subclades [7,8]. Conventionally, APV was considered to be host-species or host-order specific although many ecological and historical processes may modulate this pattern [7]. For instance, Canarypox virus (CNPV) was thought to preferentially infect passerines, while Fowlpox virus (FWPV) affected chickens and turkeys. Taxonomy of genus *Avipoxvirus* was based on this concept until recent studies showed that *Accipitriformes*, *Columbiformes*, *Otidiformes* and *Passeriformes*, can be infected by a high diversity of strains [7–10]. More in depth knowledge about the genetic diversity of APV strains is necessary because combined mutations and recombination among well characterized APV strains have been proposed as the source of the variability which may provide new strains with different pathogenicity [5]. Strain diversity in a single host may be very high, for instance, at least 17 genotypes have been described from houbara bustards, including both CNPV and FWPV genotypes [10]. Because of some APV strains may be found in several bird species the introduction of domestic birds may be a threat for wild birds, especially in isolated populations. Conversely, wild birds may be an infection source for poultry [5,11,12].

The epidemiology of avian pox infection and their distribution in natural populations is not well known because there are a number of biotic and abiotic factors that affect their distribution and prevalence [2]. A handful of studies have investigated the prevalence of poxvirus infections in wild birds around the world, most of them based on the visual observation of affected individuals. In continental birds, modal prevalence ranged from 2% to 16% in different bird species, mainly passerines [3,12–16]. The highest prevalence of pox-like lesions has been found in remote islands, such as the Canary Islands, where 50% of lesser short-toed larks (*Calandrella rufescens*) trapped around farmyards showed cutaneous lesions [4] or the Laysan albatross in Hawaii with over 88% of the individuals infected in wet years [17]. The prevalence of avian pox may be influenced by human land-use as shown in Galapagos finches [18] where prevalence increased 8-fold in agriculture areas. The authors argued that changes in innate immune function were correlated to human land-uses types and this may have determined changes in disease susceptibility. Nevertheless, other studies have obtained contradictory results about the degree of infection in urban birds [19].

Here, we studied the prevalence of cutaneous pox-like lesions and the diversity of APV strains in house sparrows, a widespread peridomestic bird, with high abundance in urban centres, around livestock and in cereal farms. *Passer domesticus* is ideal for studying the prevalence of avian pox lesions and the dynamics of a circulating pathogen because of their abundance, ubiquity and their association with human settlements. They are known to host diverse APV strains from both the CNPV and FWPV clades [8,20]. We examine whether prevalence, or strain type, varies along an urban to agricultural habitat gradient. This will provide a base level assessment for future comparison, and identify whether specific habitat types pose a higher risk for APV transmission. Sampling was carried out at different landscape types (urban, agricultural and natural) near both to farms and to the Doñana National Park in South Spain (Huelva province), which is a breeding ground and transit point for approximately 300 different species of birds. Additionally, we studied the prevalence and diversity of APV genotypes in several sites in central Spain.

## Materials and Methods

### Tissue samples and control plasmids

Two areas of the Iberian Peninsula (south and central regions) were sampled in order to determine prevalence and genetic diversity of APV in house sparrows. In central Spain (Madrid and Caceres provinces) we surveyed seven different locations between November (2012) and June (2013). In South Spain (Huelva province) 15 different sites were surveyed from July to October 2013 and from June to August 2014. Some of the sampling sites were located inside or close to protected areas, including the Doñana National Park and wetlands included in the Natura 2000 Network. In South Spain, sampling sites were geographically clustered in trios formed of an urban area (human inhabited areas in small towns or villages), an agricultural area (areas with a high presence of farm animals, usually cows, horses and/or hens) and natural areas (with low presence of humans and livestock). There were five clusters totalling 15 different sampling areas.

The annual precipitation in sampled areas in 2012 was 15% lower than historical averages (1971–2000). Precipitation in 2013 and 2014 was 10% and 5% above the historical mean respectively (AEMET; Agencia Estatal de Meteorología, Spain).

House sparrows were individually marked with unique identifying rings, weighed, aged and sexed based on plumage and skull pneumatization characteristics [21,22]. House sparrows were classed as: 1) yearlings, birds in their first year of life; 2) adults, birds in at least their second year of life and 3) birds that could not be classified, including adult birds and yearlings with a completed moult and ossified skull pneumatisation [21]. Blood samples were obtained from the jugular vein of each bird using sterile syringes. Samples from yearlings were used for the molecular identification of the sex of individuals. The volume of blood extracted depended on bird size but never exceeded 1% of avian body mass. Blood was collected in Eppendorf tubes and maintained in cold boxes in the field and at 4°C for 24 hours. After that, blood was centrifuged for 10 minutes at 4,000 rpm to separate the serum and cellular fractions. Samples were frozen at -20°C until subsequent analysis.

Birds showing evidence of potential poxvirus infection (i.e. cutaneous lesions such as hyperplastic nodules on unfeathered portions of legs, feet or head suspicious of viral infection) were recorded. Cutaneous lesions were swabbed or biopsied using sterile swabs placed in virus transport buffer (PBS, 1% of a dilution of 200mM L-glutamine, 10.000 U penicillin and 10 mg streptomycin/ml, and 0.5% gelatine) for samples obtained in the southern Spain. Swab samples from central Spain were placed in PBS and tissue samples were stored dry. Swabs were rubbed vigorously against the surface of the skin lesion. For tissue samples, a minimally invasive biopsy was excised from the edges of lesions, and great care was taken to not cause bleeding. In the infrequent event of haemostasis, digital pressure was applied to the site of the bleeding, taking care not to break the fragile avian bones. All samples were immediately frozen in portable liquid nitrogen containers or kept in cold-boxes and subsequently frozen in the laboratory on the same day. Samples were stored at -80°C awaiting molecular analysis.

Bird sampling was performed with permissions from landowners and the regional Department of the Environment (Comunidad de Madrid; Junta de Andalucía; Junta de Extremadura) and all the experimental procedures were approved by The Doñana Biological Station Ethics Committee on Animal Experimentation (CEEA-EBD) by CSIC Ethics Committee (CEC). Birds were captured in mistnets and retained until manipulation in cloth bags to keep them safe and calm. All sampled birds were released unharmed at the site of capture after manipulation.

### DNA extraction and PCR amplification

DNA extraction from cutaneous lesions and PCR amplification for APV were realized as previously described [23]. First, a multiplex PCR to detect poxvirus (APV) or papillomavirus (PV) infection was carried out using BconPVF1/BconPVR1 and P4b1060F/P4b1060R. If poxvirus was amplified, a second specific PCR, M2925-M2926 which targets *fpv167 locus*[24], was used to obtain a sequence that could be compared with published sequences. The presence of bird DNA was confirmed in all samples by amplification of a fragment of the cytochrome (cyt) *b* gene [25]. Extraction blanks, PCR negatives and standard negative controls were used in all PCRs, and were consistently negative.

First year birds for which visual sexing according to plumage characteristics was not possible, were sexed using molecular tools. Genomic DNA was extracted from the cell fraction of each blood sample using a semi-automatic Maxwell kit method (Maxwell^®^16 LEV system Research, Promega, Madison, WI) which involves an enzymatic lysis using proteinase K followed by a purification of DNA using magnetic beads that bind to DNA [26]. The sex identification test employs the primer pair CHD-P2 (5’ TCTGCATCGCTAAATCCTTT 3’) and CHD-P8 (5’ CTCCCAAGGATGAGRAAYTG 3’) [27,28] to amplify a CHD gene fragment. Each PCR reaction was performed with approximately 20 ng of genomic DNA as template using the following conditions: an initial denaturing step at 94°C for 2 min, 55°C for 30 s, 72°C for 1 min was followed by 34 cycles of 92°C for 30 s, 50°C for 30 s and 72°C for 45 s. A final run at 72°C for 5 min completed the program. The reaction products were checked by running in a 1.5% agarose gels for band visualization and sexing of each sample.

Amplicons were Sanger sequenced in both directions. The nucleotide sequences obtained were analyzed with Lasergene Suite (DNAStar, Madison, USA). Consensus sequences were compared with poxvirus sequences available using the National Center for Biotechnology Information’s Basic Local Alignment Search Tool (BLAST; http://www.ncbi.nlm.nih.gov/blast/Blast.cgi).

### Phylogenetic analyses

We determined the phylogenetic relationships between the strains of APV infecting house sparrows by building a phylogenetic tree using novel sequences from this study and published ones. We used a 448 bp fragment sequence using the simple PCR-M2925/26 set, which is homologous to sequences used in previous phylogenetic analyses of APV.

Strains differing by at least one nucleotide were downloaded from GenBank (access date 29^th^ of September of 2015) resulting in 66 P4b strains including the two strains found in house sparrows in this study. The most appropriate nucleotide substitution model was found under a Bayesian information criterion with PartitionFinder [29]: HKY+I+G. We inferred the phylogenetic relationships with a Bayesian analysis using BEAST 2.0 [30] after setting the parameters for the BEAST-run in BEAUTI 2.0 [30]. Markov Monte Carlo Chains (MCMC’s) were run for 10^9^ generations sampling every 10,000 trees using a Yule speciation prior and an estimated molecular strict clock since our data could not reject this model based on the underlying log normal distribution standard deviation (ucld.stdev) values histogram in Tracer v1.5 (http://tree.bio.ed.ac.uk/software/tracer/). All estimated sample sizes were higher than 200 and the resulting 100.000 trees were summarized in TreeAnnotator v2.1.2 (http://beast.bio.ed.ac.uk/treeannotator) after removing a 25% burn-in and are displayed in Figtree v1.4.2 (http://tree.bio.ed.ac.uk/software/figtree/).

### Statistical analyses

Prevalence of APV lesions was analyzed using General Linear Mixed Model with binomial distributed error and logit link function. Presence/absence of lesions was included in the model as the response variable, trios and site within each trio as random factors and habitat, sex, age, month and year as fixed independent factors. Only individuals with known age and sex were included in the analyses. Statistical analyses focus only on southern Spain data due to the larger dataset available and standardized sampling. Data from central Spain were used for comparison of prevalences and phylogenetic analyses. Models were fit using the GLIMMIX procedure in SAS 9.4.

## Results

### Prevalence of poxvirus-like cutaneous lesions in birds

**Fig 1:**
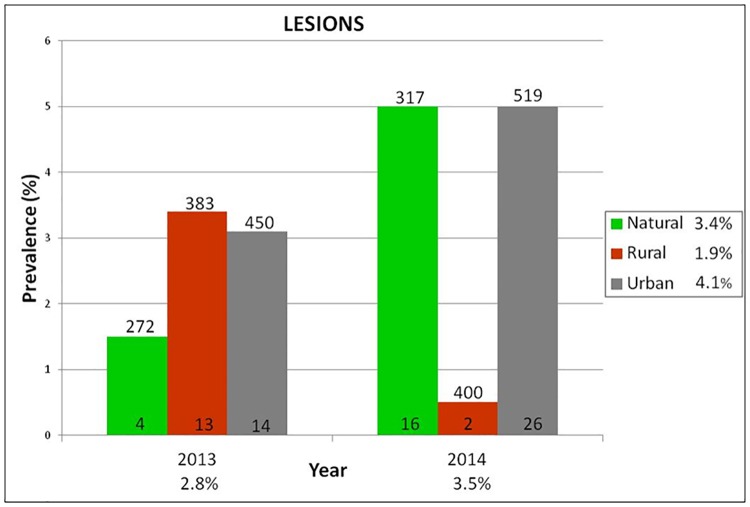
Prevalence of pox-like lesions by land-use type in the province of Huelva in 2013 and 2014. Number over bars indicate sample size, number inside bars indicate number of birds with cutaneous lesions.

We found cutaneous lesions on the unfeathered parts of the body in several house sparrows surveys carried out in Spain. Lesions were wart-like growths (0.5–6 mm in diameter) which can range in colour from yellow or white in the early stages to brown or black when the formation of crusty scabs become. Smaller lesions can be quite cryptic. Frequently these regions are bordered by erythema. The lesions were seen more frequently in lower legs and feet (S1 Fig). The mean prevalence of pox-like lesions in a survey of 2,341 house sparrows from southern Spain (Huelva) was 3.2%, ranging from 2.8% in 2013 to 3.5% in 2014 (Fig 1). Using the 1,866 first captures of house sparrows with known sex and age, no differences in prevalence were found between years (F_1,14_ = 0.39, p = 0.54), nor months (F_4,25_ = 0.89, p = 0.49). Prevalence was also similar in urban, agricultural and natural habitats (F_2,12_ = 0.30, p = 0.75), and did not differ between males and females (F_1,14_ = 0.30, p = 0.60). However, the prevalence of APV lesions in southern Spain was higher in yearling birds (F_1,14_ = 20.57, p = 0.0005).

The prevalence in birds examined in central Spain in 2012/2013 was similar to the ones from southern Spain. Ten out of 338 birds (3%) showed cutaneous pox-like lesions.

### Molecular analyses

We could only test 47 birds from the 75 individuals (63%) from southern Spain with pox-like lesions, including 33 swabs (13 from 2013 and 20 from 2014) and 14 tissue samples (all from 2014) (Table 1). APV infections were confirmed by molecular amplification in 64% of them, including 26 swabs (79%) and 4 tissues (29%). In central Spain, pox-like skin lesions were observed in ten house sparrows; swab samples were collected from all ten birds and additional tissue samples were collected from five of the ten birds. Overall, APV infection was confirmed in six of the ten house sparrows with avian pox-like skin lesions observed in central Spain. Four of the ten swabs obtained in central Spain were PCR positive, while APV infections were confirmed in each of the five tissue samples tested.

**Table 1:** Summary of total pox-like lesions, type of samples and molecular analysis of APV strains.

Location	SOUTHERN SPAIN		CENTRAL SPAIN
Years	2013	2014	2012–2013
**Total captures**	1,105	1,236	338
**Pox-like lesions**	31	44	10
**Pox-like lesions analyzed**	13	34	10
**Swabs analyzed**	13	20	10
**Tissues analyzed**	0	14	5
**Positive APV swabs (%)**	9 (69.2)	17 (85)	4
**Positive APV tissues (%)**		4 (28.6)	5
**APV positives lesions (%)**	9 (69.2)	21 (61.8)	6
**APV sequences (CNPV-PD1:CNPV-PD2)**	9 (8:1)	14 (7:7)	6 (0:6)

Global data show that the viral infection only was confirmed in 63% (36/57) of the analysed house sparrows. All samples tested negative for avian papillomavirus.

We compared 578 bp APV sequences amplified from the *fpv167* gene. We sequenced 23 amplicons from 30 samples positives for M29—from house sparrows sampled in southern Spain and six from six positives from locations in central Spain (Table 1).

**Fig 2:**
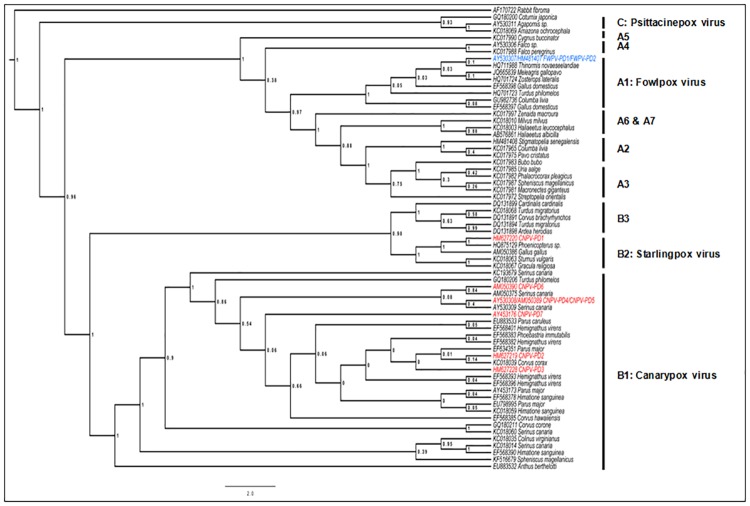
Bayesian phylogeny of DNA sequences from a 448 bp fragment of the 4b core proteins for 66 unique Avipox strains, showing posterior probability values. Avipoxvirus clades A-C following Jarmin *et al*. [8] and Gyuranecz *et al*.[7].

**Fig 3:**
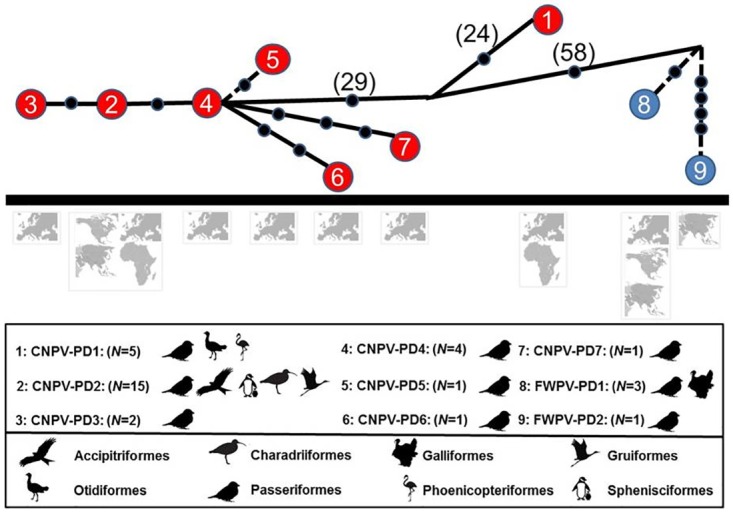
TCS haplotype network of the nine unique Avipox sequences detected in *Passer domesticus*. The maximum coverage for all nine sequences is 357 nt, though coverage for seven haplotypes is 538 nt. Canarypoxvirus (CNPV) strains are shown in red; Fowlpoxvirus (FWPV) strains are blue. Number of nucleotide substitutions are marked on the line by a solid circle, or shown in parenthesis (when numerous). The continent and avian order of detection, and number of known host species (*N* = *x*), are shown below each strain. Note that FWPV-PD1 and PD2 are identical in the 357 nt sequence, but differ at five nt sites in the extended 538 nt sequence (resolution determined only from the extended 538 nt sequence, is indicated by a dotted line). Geographic and host taxonomic information associated with these sequences is based on the extended 538 nt sequence.

Two different strains named CNPV-PD1 and CNPV-PD2 (Table 2) were identified in house sparrows from southern Spain (proportions 8:1 and 7:7 in 2013 and 2014). Only the strain CNPV-PD2 was found in house sparrows captured in central Spain. The phylogenetic analysis, which included 66 sequences of 448 nucleotides in length (some data bank sequences were shorter than the amplicon from this study), shows CNPV-PD1 and CNPV-PD2 placed in two subclades B2 and B1, respectively; both Canarypox-like viruses (Fig 2). CNPV-PD1 was the predominant sequence in southern Spain in 2013 (100% in natural and 83% in agricultural areas), but it was less common in 2014 (50% in natural and urban areas), though this variation was not statistically significant. This strain shows 100% identity to an APV from an american flamingo (*Phoenicopterus ruber ruber*) at Lisbon Zoo, Portugal (HQ875129) [a houbara bustard (*Chlamydotis undulata*) in Morocco (LK021660), a great bustard (*Otis tarda*) in Hungary (KC018066) [7,10,31] and shares 70% similarity to FWPV and CNPV. This sequence shows two amino acid insertions and one deletion at the N-terminal sequenced region compared to all other APV strains. CNPV-PD2 clusters with CNPV and has been found in Passeriformes and other bird orders in Europe and USA (Fig 3).

**Table 2:** Avipox strain diversity in *Passer domesticus*, introducing a simple descriptive nomenclature.

Sequence	Accession Number	Highest similarity	Length (bp)	Clade	Country	Reference
CNPV-PD1	HM627220	STARLINGPOXVIRUS	541	B2	Spain, Morocco	[23], this paper
CNPV-PD2	HM627219	CNPV	538	B1	Spain, Morocco	[23], this paper
CNPV-PD3	HM627228	CNPV	538	B1	Spain	[23]
CNPV-PD4	AY530308	CNPV	538	B1	Germany	[20]
CNPV-PD5	AM050389/JQ067671	CNPV	538	B1	UK	[8,32]
CNPV-PD6	AM050390	CNPV	499	B1	UK	[8]
CNPV-PD7	AY453176	CNPV	357	B1	Norway	[33]
FWPV-PD1	AY530307	FWPV	538	A1	Germany	[20]
FWPV-PD2	HM481407	FWPV	538	A1	India	[11]

**Fig 4:**
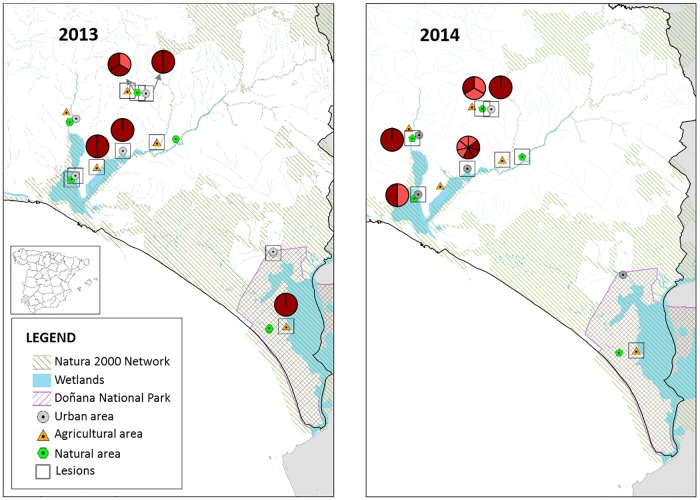
Distribution of 2013 and 2014 sampling, pox-like lesions and genotypes (CNPV-PD1, dark red; CNPV-PD2, red) in the province of Huelva. Geographical data are downloaded from http://www.ideandalucia.es/portal/web/ideandalucia.

We did not observe significant changes in the distribution of genotypes CNPV-PD1 and CNPV-PD2 between the two years of sampling or among the different areas (Fig 4), although most CNPV-PD2 was identified in the second year in natural and urban landscapes, adjacent to wetlands.

## Discussion

Most literature on APV infection describes case-reports or outbreak studies. By contrast, we focus on the prevalence of endemic disease in a single wild bird host. This is the first large-scale study of APV prevalence in house sparrows; previous studies have used more limited samples sizes [8,13,20,23,32–35]. We found a moderate prevalence (around 3%) of pox-like lesions in several areas in Spain. Similarly, in a study on 81 house sparrows captured in Hawaii authors found prevalences of 2.5% and 4.9% of active and inactive avian pox lesions, respectively [12]. Conversely, in closely related Spanish sparrows (*P*. *hispanolensis*), Smits and co-workers [4] did not find APV affected birds in the Canary Islands (n = 128). The prevalence reported here is similar to that found in blackcaps (3.7%) from the Czech Republic [36].

We did not find any significant difference in the prevalence of avian pox-like lesions in house sparrows by sex, habitat type or month during the study period. APV disease frequency has been correlated to wet seasons and rain patterns. Higher frequency has been described during winter [37], autumn in temperate climates [2] or in years with high precipitation [17]. Our results suggest that factors favouring virus transmission did not evidence temporal variation within the study period (2013–2014) in the study area. However, birds were captured during few months overlapping with the highest abundance of mosquitoes [38,39], potentially favouring virus transmission. In addition, factors including the cyclical boom in immune naïve hatch-year birds may also play a role in the observed prevalences. In fact, higher pox prevalences are more frequently reported in juveniles than in adults [2,4,16,40,41, this study]. This fact could be due to the naïve immunological status of juvenile birds or the existence of frequent asymptomatic infections in adults. Additionally, a higher mortality rate of infected juvenile birds may result in a differential prevalence between adults and juveniles.

The literature shows contradictory results about the differences of poxvirus infection in diverse habitats [18,19] Some authors have suggested prevalence may depend on parasite type and transmission methods [19]. This fact may explain our results, where no significant differences were found between habitat categories.

Most studies on the prevalence of avian pox in wild birds have relied solely on the observation of individuals with cutaneous lesions and missing digits [4,13,15] and, therefore, report a presumptive diagnosis. We confirmed viral infection by molecular analysis in 63% of all lesions tested (from approximately 50% of birds with visual lesions examined). This result fits within the values previously found in studies using tissue samples (52.1% to 82.2%) [10,34,41]. Negative results by molecular testing may be due to superficial sampling techniques; the necessity of using minimally invasive sampling of live birds may have led to some false negative results. They may also be due to lesions caused by historic infections with poxvirus, for which no viral DNA remains detectable. Alternatively, these lesions could be caused by other organisms including bacteria or mites [2,3]. Overall, >1/3 of our samples tested negative for Avipox amplification. While it is probable that these are false negative results, we concur with Parker *et al*. [3] that tests are required to confirm a diagnosis based on the macroscopic appearance of lesions.

To date, most samples used to confirm APV infection by molecular amplification are biopsies from large lesions. When lesions are very small, researchers usually avoid obtaining biopsies to avoid injuring birds. Swab collection can avoid this problem, particularly considering the good results shown in our southern Spain samples (78.8%). The lower proportion (four from ten) of positives obtained from swabs collected from cutaneous lesions in house sparrows captured in central Spain may be due to the different swab buffers used between localities. Swabs from southern Spain were conserved in virus transport buffer which likely improves the preservation of the viral genome for molecular analysis while samples from central Spain were stored only in PBS. However, this may represent biases introduced by the individual who collected the samples—note also the variation in results in positive tissue samples from southern Spain (Huelva) (28.6%) and central Spain (all of the five samples analyzed). As such best collection methods merit further investigation.

Although it is well known that the same host species can be infected with different strains of APV, the genetic diversity of strains in different hosts has been poorly studied. Currently, a total of nine APV strains have been identified in house sparrows globally, seven of which are in the canarypox clade and two in fowlpox. Three strains have been found that infect house sparrows in Spain, CNPV-PD1 to CNPV-PD3, although CNPV-PD3, highly similar to CNPV-PD2, has been identified only from a museum skin collected from central Spain in 1911 [23]. The genotype CNPV-PD1 has been detected infecting other species in the Iberian Peninsula and North Africa (Lisbon and Morocco) [10,23,31]. Although the subclade B2 consists of isolates from Sturnidae (starlings and mynahs), to date no APV has been detected in Sturnidae in Spain. The genotype CNPV-PD2 has been found in numerous wild bird species around the world. In Spain, this genotype was found in Passeriformes sampled in 2007 belonging to the genera *Cyanistes*, *Periparus* and *Sylvia* and in two museum voucher specimens dated from 1911 from the genera *Loxia* and *Passer* [23]. All samples were negative for papillomavirus but to date this viral infection has never been diagnosed in house sparrows.

In conclusion, our results reveal the active circulation of two different APV genotypes in house sparrows from Spain, with variable prevalence between age classes. In addition, our results confirm that visual inspection and molecular testing of lesions provide an incomplete estimate of APV circulation in house sparrow populations. First, the minimally invasive sampling methods employed in this study of live wild birds can involve a percentage of false negatives. Whilst the skin lesions had a characteristic appearance consistent with avian pox in this species, PCR was only able to detect avian poxvirus DNA in 63% of cases. Moreover individuals which display clinical signs of disease may not represent all individuals with current infection, nor do they capture complete information on historic infection. Further studies of seroprevalence would be extremely useful for obtaining an estimate of the real exposure to this virus in wild bird populations.

## Supporting Information

S1 Fig Macroscopic appearance of PCR confirmed Avipox lesions in two house sparrows (A and C; B: detail of lesion A). Lesions were wart-like growths (0.5–6 mm in diameter) which can range in colour from yellow or white in the early stages to brown or black when the formation of crusty scabs become. Smaller lesions can be quite cryptic.
(TIF)
