# Supplementary figures and images for: Prevalence and Genetic Diversity of Avipoxvirus in House Sparrows in Spain
Source: PLoS One. 2016 Dec 22;11(12):e0168690. doi: 10.1371/journal.pone.0168690 (PMC5179100; doi:10.1371/journal.pone.0168690)

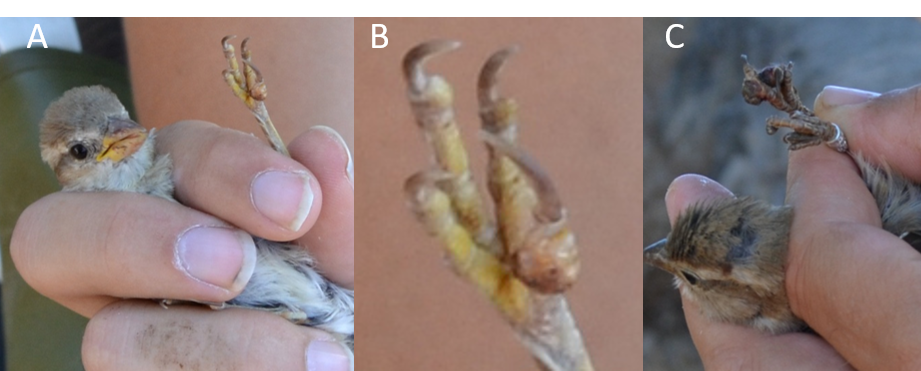

Supplement: S1 Fig — Lesions were wart-like growths (0.5–6 mm in diameter) which can range in colour from yellow or white in the early stages to brown or black when the formation of crusty scabs become. Smaller lesions can be quite cryptic. (TIF) [file pone.0168690.s001.tif]
